# Development of high-resolution melting PCR (HRM-PCR) assay to identify native fungal species associated with the wheat endosphere

**DOI:** 10.1007/s13353-020-00578-0

**Published:** 2020-08-23

**Authors:** Tomasz Cłapa, Katarzyna Mikołajczak, Lidia Błaszczyk, Dorota Narożna

**Affiliations:** 1 Department of Biochemistry and Biotechnology, Poznań University of Life Sciences, Dojazd 11, 60-632 Poznań, Poland; 2 Department of Pathogen Genetics and Plant Resistance, Institute of Plant Genetics, Polish Academy of Sciences, Strzeszyńska 34, 60-479 Poznań, Poland

**Keywords:** High-resolution melting PCR, Identification assay, fungal species, Wheat endosphere

## Abstract

Understanding the complexity and biodiversity of fungal communities associated with the wheat endosphere can facilitate the identification of novel strains that might be beneficial to the host plant. However, the differentiation and taxonomic classification of the endosphere-associated fungi with respect to various cultivars and plant organs are challenging, time-consuming, and expensive, even with the use of molecular techniques. In the present work, we describe a fast, simple, and low-cost method based on high-resolution melting PCR (HRM-PCR) for the identification and differentiation of wheat endogenous fungal isolates. Using this approach, we differentiated 28 fungal isolates, which belonged to five different genera, namely *Alternaria*, *Penicillium*, *Epicoccum*, *Fusarium*, and *Trichoderma.* Furthermore, the results of the study revealed that this method can allow large-scale screening of cultured samples.

## Introduction

Wheat is the second most widely grown cereal worldwide after rice. According to statistics, the global wheat production in 2017–2018 was 771.7 million tons (FAOSTAT, http://www.fao.org/statistics/en/, 2020). Regarding quantity and the area of cultivation, wheat is the most popular among the cereals grown in the EU, accounting for nearly half of the total production of crops (EU, Eurostat, https://ec.europa.eu/eurostat/, 2020). However, in Europe, wheat production is affected by a number of abiotic and biotic stress factors (Trnka et al. 2014). Various strategies, based on chemical, genetic, and agricultural principles, are recommended for improving the production of wheat under combined unfavorable environmental conditions. Among them, the use of biological control agents (BCAs) is the most promising and environmental-friendly approach. Fungi associated with the plant endosphere that live inside the plant tissues throughout or at least for a part of their life cycle without causing any disease symptoms their host are considered to be good candidates as BCAs (Rodriguez et al. 2009; Comby et al. 2016; Gdanetz and Trail 2017). The presence of these fungi, which are classified as endophytes, in wheat has already been demonstrated by several authors (Crous et al. 1995; Larran et al. 2002; Istifadah and McGee 2006; Lenc et al. 2015). Furthermore, some studies have demonstrated the possibility of colonizing cultivated wheat crops with endophytic fungi originating from other plant species and have reported the associated positive effects of those organisms. For example, certain endophytes were observed to reduce the susceptibility of wheat plants to insects and pathogens (Reddy et al. 2014; Keyser et al. 2016; Díaz Herrera et al. 2016), improve their heat and drought tolerance (Hubbard et al. 2012, 2014), and also promote growth (Colla et al. 2015). In light of this, understanding the complexity and biodiversity of fungal communities associated with the wheat endosphere can facilitate the identification of novel strains that might be beneficial to the host plant.

In the research carried out in recent years on fungal biodiversity, molecular approaches have been favored over morphology-based methods (Schoch et al. 2012; Blaalid et al. 2013). One of the recommended approaches based on DNA sequencing for the identification of fungal species is DNA barcoding (Schoch et al. 2012; Blaalid et al. 2013). The internal transcribed spacer (ITS) region of the ribosomal DNA (rDNA) has been proposed as the official and universal DNA barcoding marker for fungi (Schoch et al. 2012; Blaalid et al. 2013). Unfortunately, this approach is not entirely practical when a large number of samples must be analyzed, for example, while exploring the diversity of the fungi associated with wheat endosphere with respect to spatial and temporal scales (different cultivars, maturity state, different plant organs), environmental factors (controlled and field-realistic growth conditions), and agronomic manipulation (different land management conditions). Moreover, it is time-consuming and involves high costs due to sequencing. These limitations can be overcome by adopting a promising molecular method for the identification or delimitation of fungal species. One such method is high-resolution melting PCR (HRM-PCR) which screens sequence variations within amplicons by analyzing the melting temperature of the double-stranded DNA (Reed and Wittwer 2004; Erali et al. 2008; Erali and Wittwer 2010; Tahmasebi et al. 2018). In recent years, HRM-PCR has been applied for determining clinically important fungal species, such as *Candida* spp. and *Mucorales* spp. (Hrncirova et al. 2010; Decat et al. 2013; Didehdar et al. 2016; Lu et al. 2017), and for identifying and distinguishing *Fusarium oxysporum* species complex and *Aspergillus* section Nigri (Ganopoulos et al. 2012; Xanthopoulou et al. 2019).

In the present study, we attempted to develop the HRM-PCR assay for identifying the wheat endosphere-associated fungi based on differences in the melt profile of the ITS region of rDNA.

## Materials and methods

### Endophytic fungi isolation and maintenance

Endophytic fungi used in the study were isolated from roots, stems, leaves, and seeds of wheat plants cultivated in the experimental fields belonging to the Institute of Plant Genetics of the Polish Academy of Sciences in Cerekwica (Western Poland; GPS coordinates: N 52.521012, E 16.692005). A total of nine bread wheat cultivars were analyzed, which included one spring cultivar (Bombona developed by Danko Plant Breeding Ltd. Co. (Poland) and eight winter cultivars (Legenda developed by Poznań Plant Breeders Ltd., Kilimanjaro and Olivin developed by RAGT Semences Ltd. (France), Arina developed by The Swiss Federal Research Institute Changins (RAC) (Switzerland), Artist and Patras developed by Saaten Union Ltd. (Germany), Michigen Amber deposited by Plant Breeding and Acclimatization Institute (Poland), and Fregata developed by Strzelce Plant Breeding Ltd. Co. (Poland)). These plants were collected at the late milk stage (BBCH 77), placed separately in paper bags, transported to the laboratory in chilled conditions, and stored at 4 °C before processing. Within 24 h of sampling, the leaves, roots, and stems were removed from the plants and sectioned into 4- to 5-cm pieces. Seeds were threshed manually, following which seed coats were also removed. All plant fragments were surface-sterilized with 70% alcohol and 0.5% active chlorine and rinsed five times in sterile distilled water. Roots, stems, and leaves were cut with a sterile scalpel, and sections measuring 1 cm were prepared. Seeds were cut with a sterile scalpel along the crease. Then, sterilized sections of the plant organs were transferred to Potato Dextrose Agar (PDA; Oxoid™, Thermo Fisher Scientific, Waltham, MA, USA) supplemented with 50 mg mL^-1^ ampicillin and incubated at 23 °C for 1–4 weeks or until the appearance of mycelia. After incubation, putative fungal colonies were purified by subculturing three times on PDA, and then on Synthetischer Nährstoffarmer Agar (SNA; Nirenberg 1976), until visually (based on microscopic observation using a light microscope (Zeiss)) homogeneous cultures were obtained. The pure cultures were stored in tubes containing SNA at 4 °C as a working collection. For long-term preservation and in cryogenic tubes containing 20% glycerol in water (v/v) at – 80 °C .

### Identification of endophytic fungal isolates

The obtained fungal isolates were subjected to morphological and molecular-based identification. The endophytic fungi grown on PDA were studied for their culture characteristics. Microscopic analysis of morphological features was evaluated in the cultures grown on SNA by using a light microscope (Zeiss). Molecular identification was carried out based on the sequencing of the internal transcribed spacer (ITS1, 5.8S, ITS2) regions of rDNA and a fragment of the translation elongation factor 1-alpha (*Tef1*) gene, and also based on the sequencing of partial beta-tubulin 2 (*βtub*) gene depending on the fungal genus. DNA was extracted from the lyophilized mycelium of the isolates that were grown previously on the PDA medium using Wizard® Genomic DNA Purification Kit (Promega, Madison, WI, USA). The target regions of rDNA ITS were amplified by the primer sets ITS4 and ITS5 (White et al. 1990), of the *Tef1* gene was amplified by the primer sets Ef728M (Druzhinina and Kubicek 2005) and Tef1R (Kullnig-Gradinger et al. 2002), and of the *βtub* gene using primers pairs Bt2a and Bt2b (Glass and Donaldson 1995). The PCR analysis and electrophoresis were carried out under the conditions as described by Błaszczyk et al. (2016) using modification of annealing at 62 °C for the βtub gene fragment. Visibly clear PCR products were purified according to the procedure described by Gromadzka et al. (2019). The sequencing reactions of ITS, *Tef1*, and *βtub* amplicons were carried out using the ABI Prism BigDye Terminator Cycle Sequencing Ready Reaction Kit (Applied Biosystems, Switzerland) in accordance with the supplier’s instructions. Following sequencing reactions, reads were performed at the Sequencing Laboratory in the Institute of Biochemistry and Biophysics in Warsaw. The sequences were edited and assembled using the Chromas software (version 1.43, 2004; Technelysium Pty Ltd, South Brisbane, QLD, Australia) and subsequently idenifed by BLASTn analysis (NCBI, http://blast.ncbi.nlm.nih.gov/). In order to determine unique ITS haplotypes, the ITS sequences were first aligned using the ClustalW program (Thompson et al. 1994), and those exhibiting identical alleles in the ITS locus were grouped together. From each group (ITS haplotype), at least one sequence was chosen and deposited in the NCBI GenBank database. The accession numbers are detailed in Table 1.

**Table 1:** Wheat endogenous fungal strains used in the HRM-PCR analysis

Culture/strain code	NCBI GenBank accession no.	Genus
MK31	MT111904	*Epicoccum* sp.
MK50	MT111906	
MK169	MT111910	
MK138	MT111907	
MK155	MT111909	
MK146	MT111908	
MK41	MT111905	
MK218	MT111925	*Alternaria* sp.
MK141	MT111922	
MK168	MT111923	
MK136	MT111921	
MK37	MT111919	
MK35	MT111918	
MK40	MT111920	
MK133	MT112063	*Penicillium* sp.
MK164	MT112067	
MK162	MT112066	
MK158	MT112065	
MK180	MT111898	*Trichoderma* sp.
MK197	MT111900	
MK175	MT111897	
MK47	MT111933	*Fusarium* sp.
MK54	MT111934	
MK174	MT111937	
MK107	MT111935	
MK221	MT111939	
MK121	MT111936	
MK176	MT111938	

### HRM-PCR procedure

HRM-PCRs were performed using a Bio-Rad CFX Real-Time PCR Thermocycler (Bio-Rad, Poland). The total volume of 10 μL contained 1 ng of genomic DNA, 5 pM of each primer, and 5 μL of the reaction mixture with EvaGreen dye (SSoFast Supermix; Bio-Rad). The condition of the HRM-PCR reaction were as follows: initial denaturation at 98 °C for 5 min, which was followed by 30 cycles of denaturation (98 °C for 15 s), annealing (56 °C for 15 s), and extension (70 °C for 60 s); this study. For the analysis of melting curve, at the end of the amplification, one additional cycle was performed, starting with 70 °C for 2 s, following which the temperature was subsequently increased to 95 °C in 0.2 °C s^-1^ increments (HRM analyses). Data evaluation was carried out using the Bio-Rad CFX Manager Software version 1.6 and Bio-Rad Precision Melt Analysis Software version 1.1. Then, universal primers were constructed for ITS, specifically the ITS of rDNA, (ITSf 5′-AACTTTCAACAACGGATCTC-3′ and ITSr 5′-AAATGACGCTCGAACAGGCA-3′), and used in the study. The generated amplicons were 160 pb in length.

### Phylogenetic analyses

In order to confirm the results of HRM-PCR, to construct a phylogenetic tree, the analyzed sequences were used. The evolutionary history was inferred using the Neighbor-Joining method (Saitou and Nei 1987). The optimal tree had a sum of branch length equal to 1.27991273. The tree was drawn to scale, with branch lengths in the same units as those of the evolutionary distances that were used to infer the phylogenetic tree. The evolutionary distances were computed using the maximum composite likelihood method (Tamura et al. 2013) and are expressed as the number of base substitutions per site. The phylogenetic analysis involved 28 nucleotide sequences. The codon positions included were 1st + 2nd + 3rd + noncoding. All ambiguous positions were removed for each sequence pair (pairwise deletion option). The final dataset had a total of 573 positions. Evolutionary analyses were conducted using the MEGA X software (Kumar et al. 2018).

## Results and discussion

**Fig. 1:**
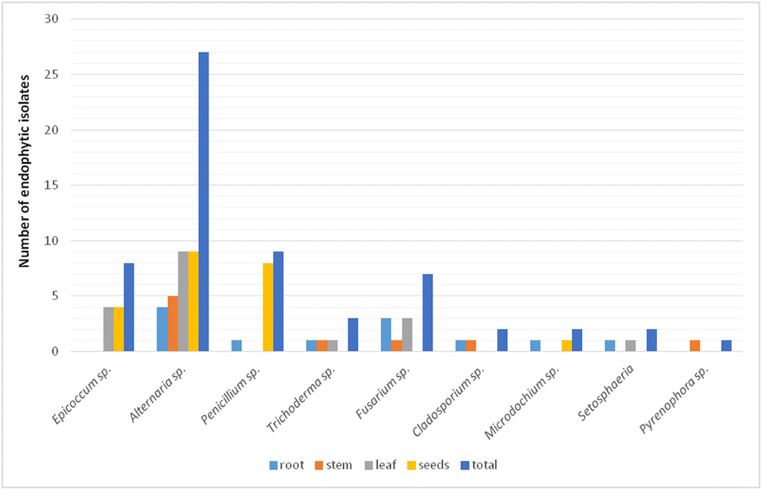
The number of wheat endophytic isolates by identified fungal genera

Overall 61 endophytic fungal isolates were obtained from all the tested wheat cultivars and plant organs (Fig. 1). They were identified by morphological characteristics, microscopic analysis, and data sequencing. *Alternaria*, *Penicillium*, *Epicoccum*, *Fusarium*, and *Trichoderma* were among the common genera present. These fungal genera have already been described as the endophytes of wheat by various authors (Crous et al. 1995; Larran et al. 2002; Comby et al. 2016). Therefore, strains belonging to these genera, representing each of the identified ITS haplotypes, were selected for the HRM-PCR analysis in the present study. As shown in Fig. 1, the genera that were represented by only individual isolates were *Cladosporium*, *Microdochium*, *Setosphaeria*, and *Pyrenophora*. Most of them were isolated from the inner tissue of wheat in the previous works (Crous et al. 1995; Comby et al. 2016). However, in the present study, these genera were excluded from the HRM-PCR analysis due to their low representativeness.

The fungal strains selected for the HRM-PCR analysis are listed in Table 1. All the samples obtained from the selected strains were amplified using the EvaGreen dye-based method using a Bio-Rad CFX Thermocycler. The specificities of the melting peaks (*T*_m_) were determined using the HRM analyses, which allowed differentiating the investigated ITS haplotypes of fungal strains. Thus, it was possible to distinguish the abovementioned fungal genera: *Epicoccum*, *Alternaria*, *Penicillium*, *Trichoderma*, and *Fusarium*. All strains were represented by a peak ranging from 79.8 to 81.2 **°**C (Table 2).

**Table 2:** Comparison of melting points

Genus	Melting temperature (°C)
*Epicoccum* sp.	80.4–80.6
*Alternaria* sp.	80.8–81.0
*Penicillium* sp.	81.2
*Trichoderma* sp.	80.2–80.4
*Fusarium* sp.	79.8–80.2

**Fig. 2:**
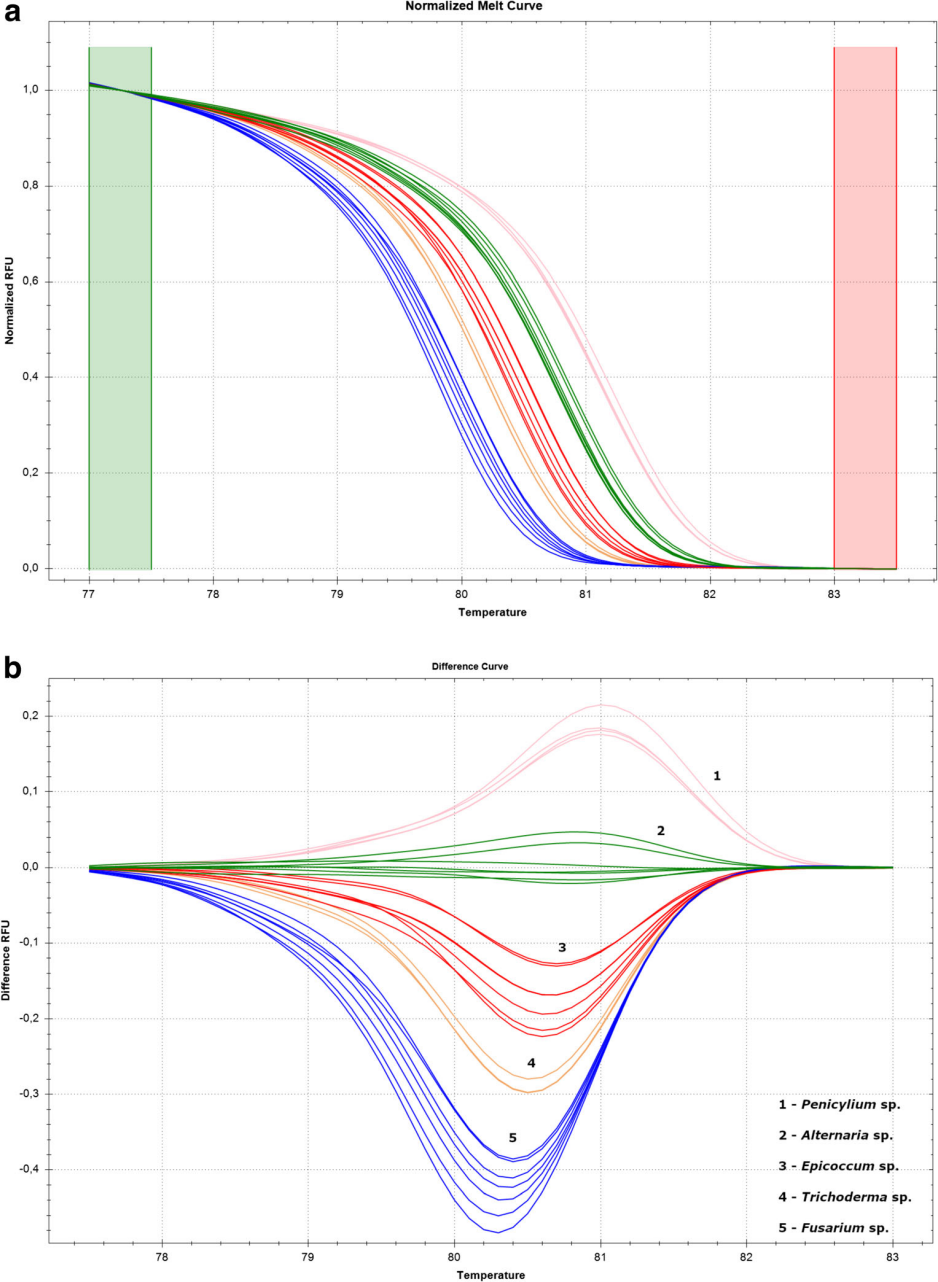
Differentiation of the fungal strains. **a** Genotyping of five fungal genera using HRM analysis. **b** Calculation of the relative ratio between the temperatures

Furthermore, in this study, we differentiated the tested fungal genera by analyzing the shape of the melting curves (Fig. 2).

**Fig. 3:**
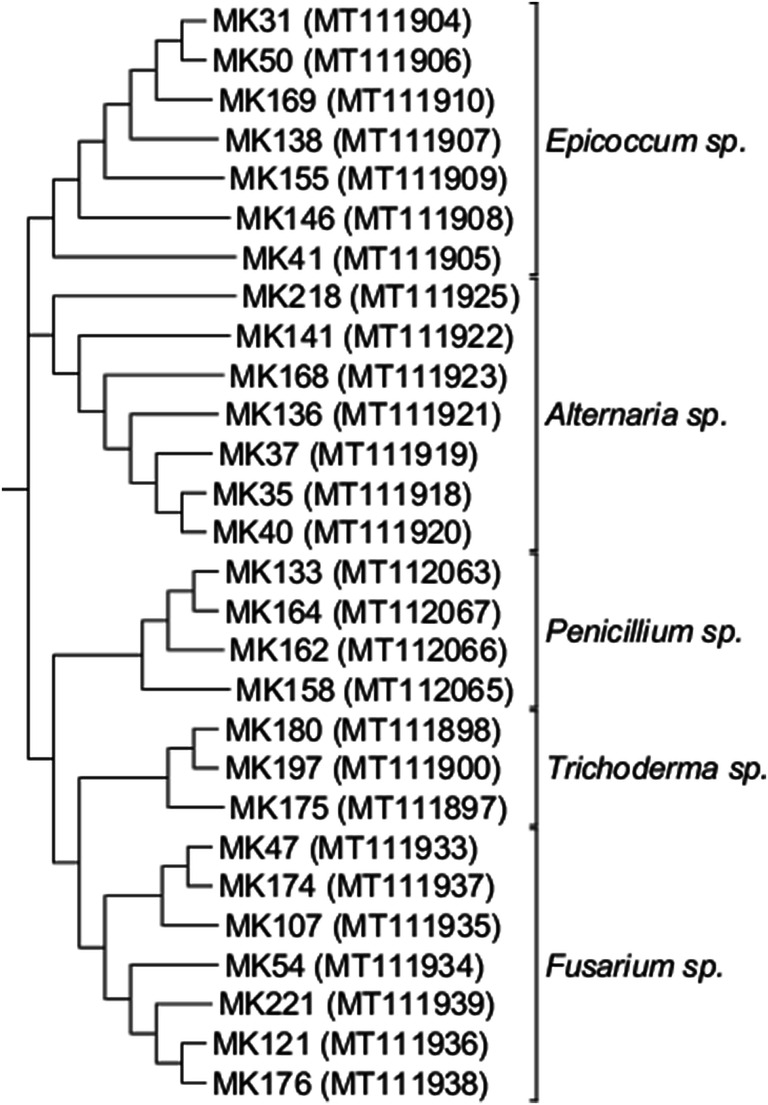
A phylogenetic tree constructed based on the analyzed ITS sequences. The numbers indicate the DNA samples of different fungi

In addition, the phylogenetic tree, which was constructed based on the sequences of the tested fungal strains, confirmed that the samples belonged to different clades (Fig. 3).

Thus far, several studies have employed the HRM technique to develop a molecular approach for the identification and discrimination of clinically relevant fungal species (Hrncirova et al. 2010; Decat et al. 2013; Didehdar et al. 2016; Lu et al. 2017), for monitoring the composition of the indoor airborne fungal contaminants (Libert et al. 2017), or for the identification of phytopathogenic fungi (Papavasileiou et al. 2016). Currently, a number of studies are focusing on exploring the wheat mycobiome (Comby et al. 2016; Gdanetz and Trail 2017) and acquiring fungal endophytes that might be beneficial to the host plant (Gdanetz and Trail 2017). The approach used in these studies indicates that it is possible to improve culture-dependent methods to identify the wheat endosphere-associated fungi. In comparison with other high-throughput technologies, such as next-generation sequencing, HRM-PCR analysis is advantageous because it is faster, simpler, and cost-effective and can accurately screen a large number of samples. Therefore, the application of this novel method for the differentiation of endophytic fungi could contribute not only to the rapid identification of new endophytes that are beneficial to plants but also to a better understanding of the complexity and diversity of the native fungal communities that are associated with the wheat endosphere.

## Conclusion

This study showed that HRM and curve analyses might be applied for large-scale screening of the cultured endophytic fungi. The universal ITS primers constructed in the study were able to distinguish five fungal genera. Real-time HRM-PCR is a cheap, sensitive, precize, and rapid assay that enables the differentiation of fungal genus. Furthermore, with the use of some standardized sequences, it allows screening a large group of isolates without sequencing fragments of ITS.
